# Meiotic Chromosome Dynamics in Zebrafish

**DOI:** 10.3389/fcell.2021.757445

**Published:** 2021-10-08

**Authors:** Yukiko Imai, Ivan Olaya, Noriyoshi Sakai, Sean M. Burgess

**Affiliations:** ^1^Department of Gene Function and Phenomics, National Institute of Genetics, Mishima, Japan; ^2^Department of Molecular and Cellular Biology, University of California, Davis, Davis, CA, United States; ^3^Integrative Genetics and Genomics Graduate Group, University of California, Davis, Davis, CA, United States; ^4^Department of Genetics, School of Life Sciences, SOKENDAI (The Graduate University for Advanced Studies), Mishima, Japan

**Keywords:** zebrafish, meiosis, chromosome, recombination, synaptonemal complex, bouquet, telomeres

## Abstract

Recent studies in zebrafish have revealed key features of meiotic chromosome dynamics, including clustering of telomeres in the bouquet configuration, biogenesis of chromosome axis structures, and the assembly and disassembly of the synaptonemal complex that aligns homologs end-to-end. The telomere bouquet stage is especially pronounced in zebrafish meiosis and sub-telomeric regions play key roles in mediating pairing and homologous recombination. In this review, we discuss the temporal progression of these events in meiosis prophase I and highlight the roles of proteins associated with meiotic chromosome architecture in homologous recombination. Finally, we discuss the interplay between meiotic mutants and gonadal sex differentiation and future research directions to study meiosis in living cells, including cell culture.

## Introduction

Meiosis is a specialized cell division program required for the production of haploid gametes and sexual reproduction. The halving of chromosome number from the diploid state to the haploid state is achieved through two sequential rounds of segregation (meiosis I and meiosis II). While meiosis II resembles mitosis by which sister chromatids are segregated to two daughter cells, meiosis I requires a specialized mechanism where homologous chromosomes (homologs) recognize each other, pair, and undergo homologous recombination to form crossovers. The combination of crossover and cohesion between sister chromatids established during prophase I is required to produce tension and alignment on the meiotic spindle in metaphase I. Errors in any of these events can lead to the formation of aneuploid gametes and are a leading cause of birth defects in humans (Hunt and Hassold, 2002; Nagaoka et al., 2012).

The events leading to crossover formation are conserved among species and include the pairing and synapsis of homologous chromosomes (Table 1). Each homolog is organized around an axial core of proteins that make up the chromosome axis. Homolog pairing is an event that relies on DNA homology to bring regions of chromosomes into close alignment, at which point the synaptonemal complex (SC) is established and spreads to join the axes end-to-end. In addition, as homologs pair, meiotic chromosomes are organized in the nucleus in a way that telomeres are held transiently together near the nuclear envelope in the bouquet configuration.

**TABLE 1:** Conservation of amino acid sequence of meiosis proteins in zebrafish.

**Function/structure**	***D. rerio***	**Size (aa)**	***M. musculus***	**Identity**	***S. cerevisiae***	**Identity**
**DSB formation**	Spo11 NP_991245.1	383	SPO11 NP_001077429.1	54%	Spo11 NP_011841.1	20%
	Iho1 NP_001313357.1	542	IHO1 NP_001128670.1	25%	Mer2 NP_012555.1	13%
**DSB repair**	Brca2 NP_001103864.2	2,874	BRCA2 NP_001074470.1	26%	–	–
	Rad51 NP_998371.2	340	RAD51 NP_035364.1	89%	Rad51 NP_011021.3	55%
	Dmc1 NP_001018618.1	342	DMC1 NP_034189.1	88%	Dmc1 NP_011106.1	54%
**Crossover resolution**	Mlh1 NP_956953.1	724	MLH1 NP_081086.2	68%	Mlh1 NP_013890.1	40%
**Meiotic cohesin**	Rad21l1 NP_001073519.1	546	RAD21L NP_001263329.1	36%	–	–
	Rec8a XP_017214597.1	595		34%		14%
			REC8 NP_064386.2		Rec8 NP_015332.1	
	Rec8b NP_001035468.1	564		32%		11%
	Smc1β XP_009296271.1	1,235	SMC1β NP_536718.1	52%	Smc1 NP_116647.1	29%
**HORMADs**	Hormad1 NP_001002357.1	356	HORMAD1 NP_001276461.1	41%		15%
					Hop1 NP_012193.3	
	Hormad2 NP_001034898.1	305	HORMAD2 NP_083734.1	38%		14%
**Synaptonemal complex**	Sycp2 XP_685048	1,569	SYCP2 NP_796165.2	28%	Red1 NP_013365.1	11%
	Sycp3 NP_001035440.1	240	SYCP3 NP_035647.2	53%	–	–
	Sycp1 NP_001112366.1	1,000	SYCP1 NP_035646.2	31%	Zip1 NP_010571.1	20%

*The zebrafish (D. rerio) protein identified by the NCBI Protein ID was aligned with the reference sequence of mouse (M. musculus) and budding yeast (S. cerevisiae) orthologs. Alignments were performed by the EMBOSS Needle Pairwise alignment program using the default settings, to calculate the percent identity. NCBI Protein IDs used for alignments are indicated with protein names.*

Mechanisms supporting meiotic pairing and recombination have been extensively studied using a number of different model organisms that include yeasts and fungi, protists, plants, and animals (Klutstein and Cooper, 2014; Zickler and Kleckner, 2015, 2016; Loidl, 2016; Hughes et al., 2018; Grey and de Massy, 2021). Nevertheless, meiosis in fish groups, which occupy more than half of vertebrate species, remains largely unknown. Several studies in the teleost zebrafish (*Danio rerio*) have indicated that homologous recombination plays an essential role in gametogenesis, where diploid cells containing 25 pairs of chromosomes (note that zebrafish does not have sex chromosomes) are segregated to haploid gametes. Furthermore, recent studies have identified key structures of zebrafish meiotic chromosomes and their functions in recombination. These studies have revealed conserved roles of such structures and provided new insights into mechanisms leading to homolog pairing and meiotic recombination.

We were motivated to write this review based on the recent enthusiasm for using zebrafish as a “new” model organism to study meiosis. Our intended audience includes researchers who are new to zebrafish and would like to incorporate zebrafish biology in their studies as well as those interested in the breadth of chromosome-based strategies to properly segregate chromosomes across species. Here we review “when and where” each chromosome event takes place within the nucleus at each prophase stage of meiosis (sections “Overview of Zebrafish Gametogenesis” and “Progression Through Meiotic Prophase I”), and compare and contrast the functions of genes studied to date with mouse and human orthologs (sections “Chromosome Structures” and “Meiotic Recombination”). Next, we highlight some of the unique aspects of the zebrafish model that relate defects in oogenesis and gonadal sex differentiation that result in female-to-male sex reversal (sections Gonadal Sex Differentiation and Sex Reversal” and “Is the Synapsis Checkpoint Absent During Oogenesis?”). We also cover advantages and disadvantages of using zebrafish as an experimental organism to study the chromosome events of meiosis compared to mice and highlight the outstanding questions where zebrafish are well-suited for study (sections “Advantages and Disadvantages of the Zebrafish Model” and “Future Perspectives”).

## Overview of Zebrafish Gametogenesis

**FIGURE 1:**
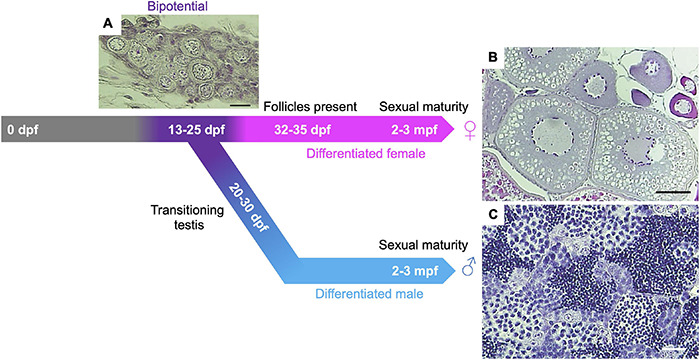
Gonadal sex differentiation and oocyte staging in zebrafish. Adapted from Blokhina et al. (2021). At 13-days post fertilization (dpf) the gonad is considered bipotential (i.e., can develop as either a testis or an ovary) and is made up of pre-follicle and early follicle stages (referred to as stage IA and IB, respectively, based on staging in Selman et al., 1993) and includes cells in meiotic prophase I. Following Stage IA oocyte formation, testis transitioning occurs ∼20–30 dpf in roughly 50% of larvae. In this case, oocytes undergo apoptosis and spermatogenesis is initiated. In animals that differentiate as females, oocytes develop further, and Stage IA oocyte production continues through adulthood. **(A)** Hematoxylin and Eosin (H&E) stained section of 24 dpf gonad. Scale bar = 20 μm. **(B)** H&E section of adult ovary. Scale bar = 100 μm. **(C)** H&E section of adult testis. Scale bar = 20 μm.

All juvenile zebrafish first develop bipotential ovaries with immature oocytes. Based on poorly understood effects of genetic and environmental contributions, immature oocytes degenerate in about half the population, leading animals to develop as males (Takahashi, 1977; Uchida et al., 2002; Maack and Segner, 2003; Rodríguez-Marí et al., 2005). The first round of oogenesis in the bipotential ovary progresses to the early follicle stage and gonadal sex differentiation occurs ∼20–25 days post fertilization (dpf) (Figure 1). Details of early oogenesis and gonad development in zebrafish are reviewed elsewhere (Selman et al., 1993; Elkouby and Mullins, 2017; Kossack and Draper, 2019). Early prophase I meiocytes can be observed in both testes and ovaries of adult zebrafish (Moens, 2006; Kochakpour and Moens, 2008; Draper, 2012; Beer and Draper, 2013). The zebrafish testis comprises lobules built of clusters of cystic germ cells, called spermatocysts, surrounded by Sertoli cells. Each spermatocyst contains a synchronously developing group of germ cells derived from a single spermatogonium (Schulz et al., 2010). A single spermatogonium undergoes nine rounds of mitotic division before entering meiosis, potentially yielding ∼512 meiocytes (spermatocytes) per spermatocyst. However, each spermatocyst has been found to contain only ∼400 germ cells at a similar sub-stage (Leal et al., 2009). Adult ovaries contain fewer prophase I meiocytes (oocytes) compared to males (Kochakpour and Moens, 2008). The majority of oocytes are located in the germinal zone, a discrete region on the surface of the ovary containing a population of germ cells <20 μm in diameter (Beer and Draper, 2013). Since female development depends on the ongoing production of oocytes (Kossack and Draper, 2019), several mutants that affect gametogenesis cause animals to undergo female-to-male sex reversal (described in section “Gonadal Sex Differentiation and Sex Reversal”).

## Progression Through Meiotic Prophase I

**FIGURE 2:**
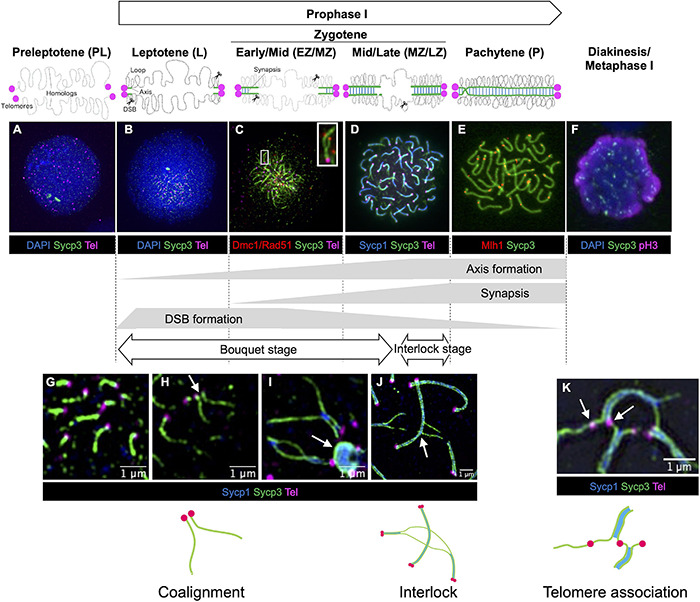
Stages of meiotic prophase I in zebrafish. Immunofluorescence staining of synaptonemal complex protein 3 (Sycp3) with telomeres (Tel), DNA (DAPI) and/or stage specific markers on zebrafish spermatocyte spreads observed by conventional immunofluorescence microscopy **(A–F)** and by super resolution microscopy **(G–K)**. Diagrams of homologous chromosome pairs (gray lines) indicate axis formation (green lines) and synapsis (blue lines) from telomeres (circles in magenta). **(A)** In the preleptotene stage, telomeres are yet to cluster and aggregates of Sycp3 are observed. **(B)** In the leptotene stage, telomeres cluster in the bouquet and axis formation as seen by the formation of Sycp3 lines immediately adjacent to telomeres. **(C)** DSBs near telomeres in leptotene and early/mid-zygotene (EZ/MZ) stages, visualized by staining DNA recombinases (Dmc1/Rad51; adapted from Takemoto et al., 2020). A region marked as a white rectangle is shown at a higher magnification at the top right. **(D)** Synapsis between homologs visualized by synaptonemal complex protein 1 (Sycp1) staining in a mid- to late zygotene (MZ/LZ) nucleus. **(E)** In the pachytene stage, axis formation and synapsis are completed and chromosomes are aligned from end-to-end. Future crossover sites are visualized by staining of MutL homolog 1 (Mlh1), which is involved in DSB repair. **(F)** A phosphohistone H3 (pH3) positive nucleus with broken Sycp3 signals. **(G)** Axes originate from telomere regions. **(H)** Coaignment between homologs, as indicated by parallel segments of axes (arrow). **(I)** Synapsis initiates between end regions. Telomeres are often seen associated with polycomplexes made up of Sycp3 and Sycp1 proteins (arrow). **(J)** End-to-end synapsis can result in interlocks where one or two chromosomes (in this case two synapsed homologs) can be trapped between another synapsed pair (arrow). Interlocks are often seen with local regions of asynapsis. **(K)** Telomere associations can persist into zygotene (shown here) and pachytene (not shown) although their numbers are reduced. Sometimes a stretch of axis can be seen spanning the ends of two unrelated chromosomes (arrow). Schematic diagrams of chromosome configurations are shown at the bottom. In **(A,B,F)** blue indicates DAPI stained DNA while in **(D,G–J)** blue indicates Sycp1 protein. **(C)** Is adapted from Takemoto et al. (2020). **(F)** Is modified from Ozaki et al. (2011). **(G–I)** Were previously published in Blokhina et al. (2019). **(A,B,D,E,J)** Replicate previously published images.

Progression of meiotic prophase I can be followed cytologically by the presence or absence of key structural components of meiotic chromatin, as well as the expression of proteins involved in meiotic processes (Figure 2). Components and properties of key meiotic chromatin structures are discussed in detail later (see section “Chromosome Structures”). Proteins involved in meiotic recombination are discussed in section “Meiotic Recombination.” Prophase I is divided into five distinct stages in most studied species–leptotene, zygotene, pachytene, diplotene, and diakinesis based on cytological characteristics (Zickler and Kleckner, 1999). Because zebrafish meiocytes are more easily obtained from males, the chromosome events of meiosis prophase I have been better described in spermatocytes, however, limited characterization of oocytes shows that the stages from leptotene through pachytene are very similar to spermatocytes (Elkouby and Mullins, 2017; Blokhina et al., 2019). Prophase I of zebrafish meiocytes can be staged in a similar manner to that of other species, but with several distinguishing characteristics described below.

### Preleptotene Stage

The preleptotene stage (also known as preleptonema) occurs shortly before or upon entry into meiotic prophase I. This stage can be identified by the presence of a few intranuclear aggregates of Sycp3 (Figure 2A; Saito et al., 2011), a structural component of the chromosome axis, similar to what is seen in male germ cells at late pre-leptotene stages in lizard (Newton et al., 2016), mouse (Scherthan et al., 1996), cattle (Pfeifer et al., 2003) and human females (Barlow and Hultén, 1998; Roig et al., 2004). In zebrafish, the bouquet has not yet formed, and telomeres are dispersed (Saito et al., 2011, 2014).

### Leptotene Stage

In most species, the leptotene stage (also known as leptonema) is defined as the stage where chromosomes are organized into chromosome axes but are not yet synapsed (Westergaard and von Wettstein, 1972). In this stage, meiotic recombination is initiated by the formation of DNA double-strand breaks (DSBs, see section “DSB Formation in Zebrafish”). In zebrafish, the chromosome axis, as seen by Sycp3 staining, appears as short stretches immediately adjacent to the telomeres (Figures 2B,G; Saito et al., 2014; Blokhina et al., 2019). At the leptotene stage, the telomeres form a prominent bouquet configuration as seen in many organisms including human males (Scherthan, 2001). In addition, about 40% of telomeres are found to be associated in clusters that contain more than two telomeres per cluster (Figure 2K), suggesting their formation is not driven solely by homology (Blokhina et al., 2019). Evidence that homolog recognition has occurred is seen by the alignment of the chromosome axes in a parallel configuration without being joined by the SC (Figure 2H). Coalignment is likely a transient state since the SC has generally been established while the axes are still very short. DNA recombinases Dmc1/Rad51, which localize to DSB sites (Figure 2C; see section “DSB Repair in Zebrafish”), and γH2AX (histone H2A.X phosphorylated at Ser 139 upon DSB formation) staining shows that these DSBs form near subtelomeric regions during the leptotene stage (Saito et al., 2011, 2014; Blokhina et al., 2019; Takemoto et al., 2020; Imai et al., 2021).

### Zygotene Stage

In the zygotene stage (also known as zygonema), synapsis of homologous chromosomes is initiated by the formation of the SC, a ribbon-like structure that bridges two homologous axes. The SC is easily visualized using antibodies to the SC component Sycp1 (Figures 2D,I). Because the initiation and lengthening of the SC among all chromosome ends is largely synchronous, the zygotene stage can be divided into early, mid- and late-stages according to the progression status of synapsis based on the total length of Sypc1 lines: leptotene to early zygotene transition (L/EZ; Sycp1 = 1–10 μm), early to mid-zygotene (EZ/MZ; Sycp1 = 10–50 μm), mid- to late zygotene (MZ/LZ; Sycp1 = 50–100 μm), and late zygotene stage (LZ; Sycp1 > 100 μm) (Blokhina et al., 2019). The telomere bouquet becomes even more prominent at the EZ stage, yet as the zygotene stage progresses, the telomeres disperse over the nucleus (Saito et al., 2014; Blokhina et al., 2019). Interestingly, even though all chromosome ends appear to be associated with their homologous partner, homologous regions at interstitial locations are typically not “paired” until they are brought together by the SC, as determined using fluorescence *in situ* hybridization (FISH) (Blokhina et al., 2019; Imai et al., 2021). This is in contrast to mice and yeast in which pairing and the initiation of synapsis occurs in interstitial regions (Zickler and Kleckner, 2015). DSBs continue to occur through the zygotene stage, and Dmc1/Rad51 foci localize on the unsynapsed axes at interstitial sites (Figure 2C). It should be noted that the bouquet configuration is readily visible in whole-mount gonad sections stained with antibodies to SC components and a telomere-probe using FISH to telomere repeats (Saito et al., 2014; Blokhina et al., 2019).

Since synapsis initiates at the ends of chromosomes and then “zippers-up” the remaining unpaired regions, it is not surprising that some chromosomal segments would become trapped between another set of homologs to form entanglements or interlocks as seen in many species (Zickler and Kleckner, 1999). Since it is not uncommon to see these structures at the late zygotene and early pachytene stages, it appears to be a distinct stage that has been termed the interlock stage. Nuclei in the interlock stage are distinguished from the late zygotene stage since they frequently contain individual pairs of chromosomes with extensive or even complete de-synapsis (Figure 2J; Blokhina et al., 2019). Since these de-synapsed chromosomes do not have evident crossovers, this stage is presumably distinct from the diplotene stage, though future work to definitively distinguish these stages is warranted. An interesting avenue of research will be to determine how interlocks and entanglements are removed and how synapsis is reestablished. Recent work in the plant *Arabidopsis* shows increased levels of entanglements in strains where expression of Topoisomerase II is knocked down using RNAi (Martinez-Garcia et al., 2018). In many species, including zebrafish, the zygotene stage is marked by substantial telomere-led movement via cytoskeletal motor proteins. It has been proposed that these movements may function in part to remove entanglements or interlocks (Koszul and Kleckner, 2009; Elkouby et al., 2016; Burke, 2018; Mytlis and Elkouby, 2021).

### Pachytene Stage

In the pachytene stage (also known as pachynema), homologous chromosomes are synapsed entirely from end-to-end (telomere-to-telomere) by the SC. In zebrafish, 25 paired chromosomes are observed at this stage (Figure 2E). Since zebrafish do not have sex chromosomes, there are no asynapsed regions that are typically associated with unpaired heteromorphic sex chromosomes as is seen in some fish species (Devlin and Nagahama, 2002; Wallace and Wallace, 2003). Occasionally, some asynapsis and separation of the axes is seen near telomeres, which can be easily observed using super-resolution microscopy, possibly indicating that the cells are transitioning to the diplotene stage (Blokhina et al., 2021). Up to the pachytene stage, staining and imaging of chromosomes using super-resolution microscopy or electron microscopy show spermatocytes and oocytes are nearly identical except that fully synapsed pachytene chromosomes are somewhat longer in females (Wallace and Wallace, 2003; Kochakpour and Moens, 2008; Blokhina et al., 2019).

### Diplotene Stage

In the diplotene stage (also known as diplonema) homologous chromosomes detach from one another through the disassembly of the SCs, but the axes remain attached at sites of crossing over referred to as chiasmata (Zickler and Kleckner, 1999, 2015). In zebrafish, definitive identification of cells in the diplotene stage (vs. late zygotene-early pachytene) has remained elusive (Wallace and Wallace, 2003; Ozaki et al., 2011). It is also possible that this stage in spermatocytes is so brief that very few cells are recovered in chromosome spread preparations. Diplotene chromosomes in oocytes undergo significant decondensation and adopt a “lampbrush” chromosome structure that can be seen in intact gonad tissue with DNA stained by DAPI. This stage is also associated with increased transcription in oocytes (Selman et al., 1993; Sumner, 2008; Elkouby and Mullins, 2017).

### Diakinesis/Metaphase I

In the last stage of prophase I, the nuclear envelope disappears and the meiotic spindle begins to form, similar to mitotic prometaphase (reviewed in Petronczki et al., 2003). Further condensation of chromosomes is observed at this stage relative to earlier prophase stages. In metaphase I, the chromosomes remain at the center of the cell (this alignment cannot be observed on chromosome spreads). Homologous chromosomes remain connected by the combination of at least one crossover and cohesion between sister chromatids. In zebrafish, spermatocyte chromosome spreads at this stage can be identified by the presence of the metaphase marker phosphohistone H3 (pH3), and fragmented Sycp3 axes (Figure 2F; Ozaki et al., 2011). These nuclei are most likely at diakinesis to metaphase I stages.

## Chromosome Structures

Recent studies have identified key structures of zebrafish meiotic chromosomes and their functions in pairing and recombination (Feitsma et al., 2007; Kochakpour and Moens, 2008; Saito et al., 2014; Blokhina et al., 2019, 2021; Takemoto et al., 2020; Zhang et al., 2020; Imai et al., 2021; Islam et al., 2021). These studies not only revealed conserved roles of such structures, but also provided new insights into how they contribute to meiotic recombination. These features are summarized below.

### Telomere Bouquet

The telomere bouquet, first described in the flatworm in 1921 by József Gelei, is a conserved feature of meiotic prophase nuclei seen in yeasts, plants, protists, and animals (Scherthan, 2001). In the bouquet, telomeres cluster to one side of the nucleus and the extended chromosomes appear as stems in a bouquet of flowers. The telomere bouquet is a prominent feature of the zebrafish meiotic nucleus and provides a means to easily visualize the earliest contacts formed between homologous chromosomes (Wallace and Wallace, 2003; Saito et al., 2014; Elkouby et al., 2016; Blokhina et al., 2019). The timing of the bouquet varies among species (Zickler and Kleckner, 1998), but in zebrafish it is seen in the leptotene stage and extends into early zygotene. By late zygotene, cells begin to exit the bouquet and telomeres are dispersed, yet still attached at the nuclear envelope. It is likely that the telomeres are attached to components of the LINC complex, which forms a protein bridge linking telomeres to cytoskeletal motor proteins, but this has not been tested (Burke, 2018). Three findings support this model though. First, α− and γ− tubulin localize to the region where telomeres are clustered in the bouquet (Saito et al., 2014); second, treatment of oocytes with the microtubule inhibitor nocodazole results in the dispersal of telomeres (Elkouby et al., 2016); and third, live-imaging of oocytes shows a considerable degree of motion at this stage (Mytlis and Elkouby, 2021).

In zebrafish, components of the meiotic chromosome axis are assembled immediately adjacent to the telomeres in the leptotene stage (Figure 2G), suggesting that some component of the shelterin complex or meiosis-specific associated proteins, such as Terb1 and Terb2 or Majin may be involved in seeding the initiation of axis biogenesis (de Lange, 2005; Shibuya et al., 2014, 2015). In *Terb1^–/–^*, *Terb2^–/–^*, and *Majin^–/–^* mice, telomere attachment to the nuclear envelope is disrupted (Daniel et al., 2014; Shibuya et al., 2014, 2015; Pendlebury et al., 2017; Dunce et al., 2018; Wang et al., 2019, 2020). These proteins are conserved among metazoans, so they likely play a similar role in zebrafish (da Cruz et al., 2020).

Telomere associations represent some of the first contacts between chromosomes (Blokhina et al., 2019). In the leptotene stage, up to 50% of chromosome ends are engaged with one or more telomeres and this number decreases as prophase I progresses to ∼6% in the pachytene stage. It is possible that the process of synapsis itself physically disrupts telomere associations between unrelated chromosomes since the number of engaged ends remains high in the absence of synapsis (Blokhina et al., 2019), although the process may also be regulated and the release is coordinated with other events. Since telomere associations can involve multiple chromosomes, they likely do not depend on DNA homology, however, it is not known to what extent telomere associations are a prerequisite of *bona fide* homolog pairing.

In zebrafish, pairing begins exclusively at the ends of each homologous chromosome pair before the chromosome axis is fully formed. Prior to synapsis, coaligned axes less than 0.5 μm apart mark the earliest detectable signs of pairing at the leptotene stage prior to the appearance of the SC (Figure 2H), but this state is only transient since coaligned axes with no associated SC are relatively rare compared to synapsed ends (Figure 2I; Blokhina et al., 2019). Once paired, synapsis is initiated near the ends and extends toward the middle of the chromosome, lagging slightly behind the elongating axes (Wallace and Wallace, 2003; Ozaki et al., 2011; Saito et al., 2011, 2014; Blokhina et al., 2019). This feature is shared with spermatogenesis in humans and mice (Brown et al., 2005; Bisig et al., 2012; Pratto et al., 2014; Gruhn et al., 2016). Pairing and synapsis in zebrafish depends on the formation of meiotic DSBs (Blokhina et al., 2019; Takemoto et al., 2020). The localization of DSBs near subtelomeric regions in the bouquet are consistent with the skew of the positions of Mlh1 foci and crossing over by genetic mapping (Postlethwait et al., 1998; Kochakpour and Moens, 2008; Anderson et al., 2012).

Interestingly, the telomere bouquet is configured adjacent to the Balbiani body (Bb), a membraneless organelle that sits outside the nucleus of oocytes and comprises mRNA protein granules (mRNP) and embryonic patterning factors. Since the Bb is the site of the oocyte vegetal pole, the bouquet configuration and oocyte patterning appear to be functionally coupled (Marlow and Mullins, 2008; Abrams and Mullins, 2009). This is supported by experiments showing disruption of microtubules disrupts both the Bb and the bouquet (Elkouby et al., 2016). It thus appears that oogenesis in zebrafish has evolved to take advantage of a conserved feature of meiosis as a mechanism to break cell symmetry (Elkouby et al., 2016).

### Chromosome Axis

**FIGURE 3:**
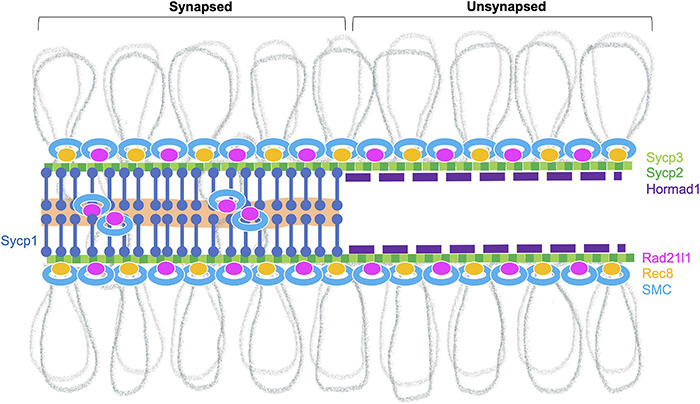
Meiotic chromosome axis structures. The synaptonemal complex comprises the lateral elements (LE, Sycp2 and Sycp3) that run along the lengths of homologous chromosomes joined by a central region that contains the transverse filament (Sycp1) and a central element (a region indicated in orange). Chromosomes joined by this tripartite structure are considered “synapsed.” Prior to synapsis, the LE is referred to as the axial elements (AE) where chromatin is organized into loops that are serially attached to the axis. The chromosome axis is made up of cohesins, the axial element proteins and HORMA-domain proteins. In zebrafish, axis localization has been observed for the cohesin components Smc3, Smc1β, and Rad21l1, the axial element proteins Sycp2 and Sycp3, and Hormad1. Localization of Rec8a/b (there are two paralogs in zebrafish) and Hormad2 (not shown) remains to be determined. Homologous chromosome pair at the ends as seen by the coalignment of axial elements. While the Rec8 cohesin complex most likely links sister chromatids together, it is not clear what DNA sequences are associated with Rad21l1 complex, however, it could play a role similar to the COH-3/4 cohesin complexes that enable the formation of asymmetric chromosome loops in *C. elegans* (Wolger et al., 2020).

Meiotic chromosomes are organized in a special higher-order structure that consists of chromatin loops anchored along proteinaceous axes (Zickler and Kleckner, 2015). The meiotic chromosome axis comprises meiosis-specific cohesin complexes, components of the SC, and HORMA-domain proteins (Figure 3; Grey and de Massy, 2021). In zebrafish, nearly all chromosome structures studied to date appear to originate from telomeres in the bouquet, including several cohesin proteins (Rad21l1, Smc3, Smc1β), the SC proteins (Sycp1, Sycp2, Sycp3), and Hormad1 (Ozaki et al., 2011; Saito et al., 2011, 2014; Takemoto et al., 2020; Blokhina et al., 2021; Imai et al., 2021; Islam et al., 2021).

DNA loop lengths can vary in different organisms. For example, the average size in mice is a few hundred kb and ∼28 kb in *S. cerevisiae* (Grey and de Massy, 2021). In zebrafish, total SC length (= axis length in the pachytene stage) per nucleus has been reported from several labs and ranges from 166 to 260 μm (Traut and Winking, 2001; Wallace and Wallace, 2003; Moens, 2006). Since the zebrafish genome size is ∼1.4 Gb (the latest zebrafish genome assembly, GRCz11), a rough estimation of SC length per Mb DNA is ∼153 nm SC/Mb DNA. Similarly, total autosomal SC length has been measured in several mouse strains and ranges from 150 to 163 μm (Vranis et al., 2010). In mice, this gives roughly ∼74 nm SC/Mb DNA for 2.1 Gb of all autosomal DNA (GRCm39). If we assume that loop density along the SC is similar among species (Zickler and Kleckner, 1999), loop size in zebrafish might be smaller than mouse, though it remains to be determined.

#### Cohesins

Following premeiotic DNA replication in S-phase, sister chromatids must remain together until meiosis II. Sister chromatid cohesion relies on ring-like protein complexes called cohesins. Cohesins are composed of two SMC (structural maintenance of chromosomes) proteins that form the ring and the additional protein subunits, kleisin and SA, that close the ring. The meiotic cohesin complex consists of four core subunits. The ring comprises SMC1β and SMC3, a kleisin subunit (RAD21L, REC8 or RAD21), and the SA subunit STAG3 (Rankin, 2015; Ishiguro, 2019). Several orthologs of these proteins have been characterized in zebrafish as summarized below (Takemoto et al., 2020; Blokhina et al., 2021; Islam et al., 2021).

SMC1β/Smc1β is one of two vertebrate SMC1 proteins: SMC1α and SMC1β. Mouse SMC1β has been shown to be a meiosis-specific component of the cohesin complex, while SMC1α is specific to somatic cells (reviewed in Ishiguro, 2019). Mouse *Smc1*β is essential for meiosis in both sexes and its mutation leads to meiotic arrest at the pachytene stage in males and at metaphase II in females (Revenkova et al., 2004). Zebrafish Smc1β is also required for both spermatogenesis and oogenesis and is expressed in both testis and ovaries (Islam et al., 2021). *smc1*β*^–/–^* spermatocytes enter the leptotene to early zygotene stages before undergoing apoptosis. Prior to arrest, short Sycp3 lines form near the bouquet, yet undergo only limited extension and do not assemble a full SC. DSBs are located near telomeres in the bouquet in this mutant suggesting that early pairing may take place but is quickly lost as cells undergo apoptosis. Smc3 lines are also not seen in this mutant as would be expected if Smc1β is required to make the meiosis-specific cohesin.

Rad21l1, a meiosis-specific cohesin subunit, is the zebrafish homolog of mouse RAD21L and human RAD21L1 (Gutiérrez-Caballero et al., 2011; Ishiguro et al., 2011; Polakova et al., 2011; Blokhina et al., 2021). Zebrafish Rad21l1 is expressed from the leptotene stage through the pachytene stage (Takemoto et al., 2020; Blokhina et al., 2021). At the leptotene stage, Rad21l1 colocalizes with the chromosome axes at the ends of chromosomes and is also found throughout the nucleus as dispersed foci. Rad21l1 loads onto axes during extension and is also found associated with the central region of the synaptonemal complex once homologs are synapsed in both spermatocytes and oocytes. At the pachytene stage, Rad21l1 remains on both the axes and central region. The dispersed foci that are observed in the leptotene and early zygotene stages have largely disappeared by late zygotene, likely having been incorporated during synapsis. In mouse, a similar staining pattern is seen except that the ortholog RAD21L transiently disappears from synapsed chromosomes at the pachytene stage (Gutiérrez-Caballero et al., 2011; Ishiguro et al., 2011; Lee and Hirano, 2011; Lee, 2013; Biswas et al., 2016; Rong et al., 2016). Spermatocytes of *rad21l1^–/–^* zebrafish show normal telomere bouquet formation and overall normal homolog pairing, yet small stretches of axes of pachytene chromosomes remain partially unsynapsed (Blokhina et al., 2021). Moreover, the *rad21l1^–/–^* males are fertile and give rise to normal progeny indicating normal recombination and chromosome segregation has occurred. This phenotype is in striking contrast to *Rad21l^–/–^* mice where the telomere bouquet, synapsis, and recombination are all severely compromised in spermatocytes (Gutiérrez-Caballero et al., 2011; Ishiguro et al., 2011, 2014; Llano et al., 2012; Biswas et al., 2016). The *rad21l1^–/–^* zebrafish populations develop almost entirely as males (see section “Gonadal Sex Differentiation and Sex Reversal”) so its localization in oocytes has not been tested (Blokhina et al., 2021).

#### Sycp2 and Sycp3

Sycp2 and Sycp3 are AE proteins conserved among metazoans (Fraune et al., 2012, 2014). SYCP2 was first identified in rats as a component of the LEs of the SC and shows some similarity to the yeast axis protein Red1 (Offenberg et al., 1998). Mouse SYCP2 directly interacts with SYCP3 through its internal coiled-coil domain (Yang et al., 2006; West et al., 2019), and the deletion of the SYCP3-interacting domain of SYCP2 leads to mislocalization of SYCP3 as aggregates (Yang et al., 2006). In zebrafish spermatocytes, Sycp3 appears as a few intense foci at the pre-leptotene stage, whereas Sycp2 is not yet observed on chromosome spreads (Ozaki et al., 2011; Takemoto et al., 2020). It is unknown whether Sycp3 at this stage forms aggregates or polycomplexes, which are observed with SC components in different species (reviewed in Hughes and Hawley, 2020). At the leptotene stage, Sycp3 starts to form short stretches upon the appearance of Sycp2 signals, immediately adjacent to telomeres (see above) (Takemoto et al., 2020). In *sycp2^–/–^* spermatocytes, Sycp3 remains as aggregates similar to what is observed in the wild-type pre-leptotene stage (Takemoto et al., 2020). Therefore, Sycp2 expression is essential for Sycp3 loading to the chromosome axis, suggesting that the zebrafish Sycp2 and Sycp3 may form a filament structure together, similar to their mammalian orthologs (Yang et al., 2006; West et al., 2019). Because Sycp3 loading also initiates adjacent to telomeres in *spo11^–/–^* zebrafish spermatocytes (Blokhina et al., 2019), DSB formation does not trigger this process. SYCP2 is observed as short fragments or dots colocalizing with telomeres in *Sycp3^–/–^* mutant mice and may have telomere specific binding properties (Liebe et al., 2004). It is possible that this function of Sycp2 in zebrafish is responsible for initiating axis formation solely at telomeres.

In *sycp2^–/–^* spermatocytes, Sycp1 is observed as aberrant filaments that are not homogeneous in length, and their numbers are decreased compared to those in wild-type nuclei (Takemoto et al., 2020). The majority (∼75%) of the aberrant Sycp1 filaments do not colocalize with telomere foci, while they are costained with Rad21l1. Thus, Sycp1 seems to be ectopically loaded onto chromatin in the absence of Sycp2. Along with the synaptic defects, homologous pairing is also defective in *sycp2^–/–^* spermatocytes as indicated from increases in numbers of telomere foci and dissociation of an interstitial locus (Takemoto et al., 2020).

#### Hormad1 and Hormad2

Members of the meiotic HORMAD family share the evolutionarily conserved HORMA (Hop1, Rev7, Mad2) domain (reviewed in Rosenberg and Corbett, 2015; Grey and de Massy, 2021). In many species, the meiotic chromosome axis contains HORMAD proteins: yeast Hop1, mammalian HORMAD1, and HORMAD2, plant ASY1 and ASY-2, and nematode HIM-3, HTP-1, HTP-2, and HTP-3. Mouse HORMAD1 is essential for both male and female fertility (Shin et al., 2010; Daniel et al., 2011), while HORMAD2 mutations cause sterility in males only (Kogo et al., 2012; Wojtasz et al., 2012). In mice, HORMAD1 appears at the preleptotene stage as distinct foci co-localizing mostly with the cohesins REC8 and RAD21L (Fujiwara et al., 2020), and form short stretches in the zygotene stage (Wojtasz et al., 2009; Fukuda et al., 2010). In zebrafish, the Hormad1 ortholog appears as punctate or short filamentous signals that partially colocalize with Sycp2-stained axes in the leptotene stage, localizes with unsynapsed axes during the zygotene stage, yet disappears in regions where synapsis occurs through the pachytene stage (Imai et al., 2021). This localization pattern on unsynapsed axes resembles that of mouse HORMAD1 (Wojtasz et al., 2009; Fukuda et al., 2010). Zebrafish knockouts of *hormad1* and *hormad2* have not been characterized.

### Synaptonemal Complex

The SC comprises two lateral elements (LEs, axes of each homolog) and a ∼100 nm wide central region that consists of transverse filaments (TFs) interacting with each LE at one side and with the central elements (CEs) at the other side (reviewed in Fraune et al., 2012; Cahoon and Hawley, 2016). Formation of LE precedes loading of TFs, and at this stage, LEs are generally mentioned as axial elements (AEs). Although the amino acid sequences of SC components proteins are poorly conserved among species (Table 1), the basic structure of the SC is highly conserved. In mammals, eight SC components have been identified: AE/LE proteins SYCP2 and SYCP3, the transverse filament protein SYCP1 (Meuwissen et al., 1992), and central element proteins SYCE1, SYCE2, SYCE3, SIX6OS1, and TEX12 (Costa et al., 2005; Hamer et al., 2006; Schramm et al., 2011; Gómez et al., 2016). Orthologs of these proteins are found in zebrafish, except for SIX6OS1, which is possibly absent in bony fish (Gómez et al., 2016). Sycp1 staining has been used to follow SC formation (synapsis) in zebrafish meiosis (Moens, 2006; Saito et al., 2014; Blokhina et al., 2019; Imai et al., 2021). The Sycp1 loading is promoted by DSB formation, since no or a few short fragments of Sycp1 are observed in *spo11^–/–^* zebrafish meiocytes (Blokhina et al., 2019). In *sycp2^–/–^* zebrafish, Sycp1 forms aberrant filaments that are heterogeneous in length, with the majority located away apart from telomeres (Takemoto et al., 2020) indicating a role for Sycp2 in SC assembly. In contrast, Sycp1 is not required for the AE/LE formation (Imai et al., 2021).

## Meiotic Recombination

### Meiotic Recombination: A General View

**FIGURE 4:**
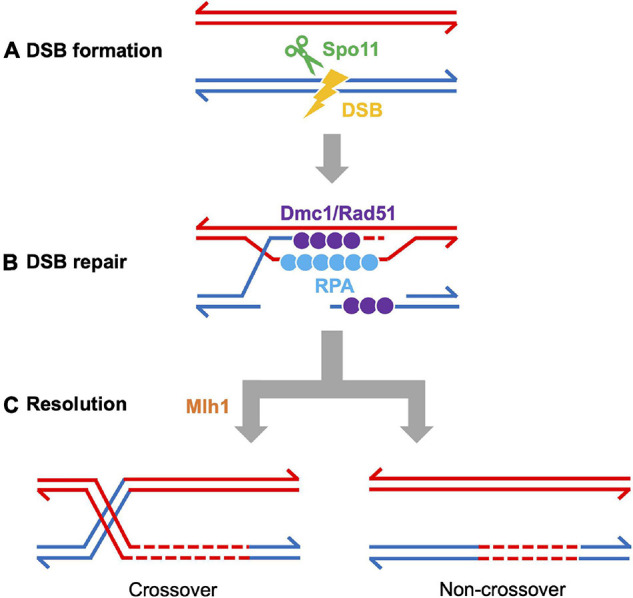
Meiotic recombination pathway showing steps mediated by proteins described in the text. Adapted from Baudat et al., 2013. Red and blue lines represent double strand DNAs of maternal and paternal origins. Broken lines indicate newly synthesized DNA strands during DSB repair. **(A)** DSB formation requires the topoisomerase-like protein Spo11. **(B)** The repair of meiotic DSBs involves strand exchange mediated by the ssDNA binding complex RPA, the recombinases Dmc1 and Rad51, and Brca2. **(C)** Recombination intermediates are resolved to form either crossover or non-crossover products. Crossover resolution involves Mlh1. Other proteins have also been shown to act in meiotic recombination in mammals (reviewed in Baudat et al., 2013) and in budding yeast (reviewed in Yadav and Claeys Bouuaert, 2021). Presented proteins have been studied in zebrafish by mutation and/or cytological analyses. It has not been definitively shown that FANCL/Fancl acts at these stages in both mouse and zebrafish.

Meiotic recombination is initiated by the formation of programmed DSBs catalyzed by the meiosis specific endonuclease SPO11 (Bergerat et al., 1997; Keeney et al., 1997), together with accessory DSB proteins (reviewed in de Massy, 2013). SPO11 is widely conserved among eukaryotes, including zebrafish (Malik et al., 2007; Sansam and Pezza, 2015; see section “DSB Formation in Zebrafish”). To ensure the proper segregation of homologous chromosomes at meiosis I, a subset of DSBs are repaired by homologous recombination to form at least one crossover per homolog pair (Figure 4). The recombination process can be broken up into 3 main stages (reviewed in Hunter, 2015): (1) DSB sites are processed by endo- and exonucleases that generate 3′-overhang single-stranded DNA (ssDNA) tails; (2) strand invasion of ssDNA is catalyzed by recombinases and co-factors; (3) recombination intermediates are processed to form either crossover or non-crossover products. A subset of DSBs are repaired using the sister chromatid in yeast (Goldfarb and Lichten, 2010; Lam and Keeney, 2014). Measuring these events in metazoans has remained elusive, yet recent work in *C. elegans* has shown they occur, though at reduced levels (Almanzar et al., 2021; Toraason et al., 2021).

Several DSB repair proteins are used to visualize the recombination process. For example, foci of DMC1 and RAD51 recombinases loaded to ssDNA are used to estimate the number and location of DSBs. Localization of MLH1 (MutL protein homolog 1), a protein required for crossover resolution, indicates putative crossover sites (Figure 2E). These proteins are also conserved among vertebrates (Table 1) and have been used to characterize the recombination process (Kochakpour, 2009; Sansam and Pezza, 2015). One challenge in the field has been to find antibodies to human and mouse proteins that cross-react with zebrafish proteins. Examples of antibodies that work (or do not work) in our hands are reported elsewhere (Blokhina et al., 2020).

### Double-Strand Break Formation in Zebrafish

Like other species, zebrafish Spo11 is essential for DSB formation, since γH2AX signals and Dmc1/Rad51 localization are abolished in *spo11^–/–^* spermatocytes (Blokhina et al., 2019; Takemoto et al., 2020). In mice, the RMMAI complex (REC114-MEI4-MEI1-ANKRD31-IHO1) is recruited onto chromosome axis via the interaction of HORMAD1 and IHO1 (Libby et al., 2003; Kumar et al., 2010, 2018; Stanzione et al., 2016; Papanikos et al., 2019; Boekhout et al., 2019). Since all of the RMMAI complex proteins, aside from ANKRD31, are essential for DSB formation in mice, this complex is thought to promote DSB formation catalyzed by the SPO11-TOP6BL complex (Libby et al., 2003; Kumar et al., 2010, 2018; Robert et al., 2016; Stanzione et al., 2016). These accessory DSB proteins exist in zebrafish, at least at the genome sequence level, though their functional conservation remains to be elucidated. Zebrafish Iho1 appears on axes at leptotene through early zygotene stages and localizes on unsynapsed axes as punctate foci or short stretches during the zygotene stage and disappears from axes at the pachytene stage (Imai et al., 2021). Iho1 foci are observed in *spo11^–/–^* zebrafish spermatocytes (Imai et al., 2021), indicating that Iho1 loading to the axis occurs prior to DSB formation, as observed in mice (Dereli et al., 2021).

In mice and humans, PRDM9 binding sites shape the DSB landscape (Baudat et al., 2010; Myers et al., 2010; Parvanov et al., 2010; Brick et al., 2012; Pratto et al., 2014). PRDM9 is a DNA-binding, zinc finger, histone methyltransferase that determines the location of recombination hotspots in a number of mammalian species (Paigen and Petkov, 2018). Zebrafish Prdm9 is missing several domains and is unlikely to play a role in DSB localization (Baker et al., 2017). However, it is an outlier since most bony fish appear to have a functional ortholog (Baker et al., 2017). Other examples of vertebrates lacking a functional Prdm9 gene, include sticklebacks (Shanfelter et al., 2019), birds and crocodiles (Oliver et al., 2009; Singhal et al., 2015), canids (Oliver et al., 2009; Muñoz-Fuentes et al., 2011), and amphibians (Baker et al., 2017). *Prdm9* knockout mice in specific genetic backgrounds and a rare healthy PRDM9 human female can reproduce normally (Muñoz-Fuentes et al., 2011; Singhal et al., 2015; Narasimhan et al., 2016; Mihola et al., 2019). In sticklebacks, birds, and dogs, recombination hotspots are typically found in promoter regions (Auton et al., 2013; Singhal et al., 2015) and is also seen in budding yeast (Lam and Keeney, 2015), which also lacks Prdm9. It is plausible that DSBs in zebrafish may be directed at promoter regions as well.

Notably, γH2AX and Dmc1/Rad51 signals in zebrafish spermatocytes appear in proximity to telomeres at the leptotene to early zygotene stages, indicating that DSBs first occur at DNA sequence near the ends of chromosomes (Saito et al., 2011; Sansam and Pezza, 2015; Blokhina et al., 2019; Takemoto et al., 2020). How DSBs are induced near telomeres in zebrafish is unknown, but recent work in human males points to early DNA replication timing influencing the skew of both DSBs and crossovers to the ends of chromosomes (Coop et al., 2008; Pratto et al., 2014, 2021). Since zebrafish do not appear to have a functional PRDM9 gene, it will be interesting to test if trimethylated H3K4 and/or H3K36 modifications, the marks created by PRDM9, are found to be enriched near telomeres. Understanding how the sites of DSBs are determined in zebrafish will likely provide insights into human spermatogenesis (Blokhina et al., 2019).

In zebrafish, each nucleus at the leptotene to early zygotene stages has an average of ∼80 Dmc1/Rad51 foci, though the number fluctuates from cell to cell (Imai et al., 2021). While this is fewer than the ∼200–400 Dmc1/Rad51 foci observed at the leptotene stage in mice and humans (Reviewed in Baudat and de Massy, 2007), it remains to be elucidated whether this lower number reflects fewer DSBs overall, rapid turnover of Dmc1/Rad51 at DSB sites, and/or an extended time window when DSBs can form.

### Double-Strand Break Repair Zebrafish

In zebrafish, Dmc1/Rad51 foci localize to chromosome axes, and based on their proximity to telomeres in the bouquet, they are most likely involved in homolog pairing (Blokhina et al., 2019; Takemoto et al., 2020; Imai et al., 2021). It is not known, however, if zebrafish Rad51 and Dmc1 localize to the same or different sites and/or play distinct roles in DSB repair, as reported in several species (Shinohara et al., 1997; Pittman et al., 1998; Yoshida et al., 1998; Howard-Till et al., 2011; Steinfeld et al., 2019; Slotman et al., 2020). In mice, RPA (Replication Protein A complex) is required for DMC/RAD51 recruitment and strand invasion as well as efficient meiotic crossover formation (Sugiyama et al., 1997; Soustelle et al., 2002; Shi et al., 2019; Hinch et al., 2020). Zebrafish RPA likely plays similar roles in meiotic recombination, since it is observed as punctate foci or short stretches mostly at chromosome ends in the leptotene and zygotene stages, and the majority of these signals disappear at the pachytene stage (Moens, 2006; Takemoto et al., 2020). Zebrafish Brca2 (Breast cancer 2) and Fancl (Fanconi anemia complementation group L) could also be involved in meiotic DSB repair, since their mutation causes defects in gametogenesis (see section “Gonadal Sex Differentiation and Sex Reversal”) (Rodríguez-Marí et al., 2010; Shive et al., 2010; Rodríguez-Marí and Postlethwait, 2011).

### Crossover Resolution in Zebrafish

MLH1–MLH3 function together to create the MutLγ nuclease that functions to bias the repair of Spo11-induced DSBs to form crossover products (Rogacheva et al., 2014; Hunter, 2015; Cannavo et al., 2020). As seen in most species, Mlh1 foci localize to the SC of pachytene chromosomes and mark the sites of putative crossovers (Hunter, 2015). In zebrafish, there are on average ∼1.0 Mlh1 foci per homologous pair in spermatocytes and ∼1.6 in oocytes in the pachytene stage (Moens, 2006; Kochakpour and Moens, 2008). Mlh1 localization shows extensive sex differences: Mlh1 foci are mostly located at distal regions of the SCs in males but tend to be more evenly distributed in females (Kochakpour and Moens, 2008). The sex-differences are also supported by zebrafish recombination mapping that predict male crossovers are skewed toward the ends of chromosomes and female crossovers are more evenly distributed along chromosomes (Postlethwait et al., 1998; Singer et al., 2002; Anderson et al., 2012). It is not known how crossover landscapes are established in females, yet it should be noted that synapsis initiates near the telomeres in the bouquet in both male and female zebrafish (Blokhina et al., 2019). Rad51 foci have also been observed at interstitial regions of pachytene chromosomes and are potential sites of crossing over (Blokhina et al., 2019; Imai et al., 2021). It will be of future interest to understand how DSBs could result in different crossover outcomes between male and female zebrafish.

### The Role of the Synaptonemal Complex in Meiotic Recombination in Zebrafish

In *sycp2^–/–^* spermatocytes, the number of Dmc1/Rad51 foci are dramatically reduced compared to wild-type (Takemoto et al., 2020). It is possible that the *sycp2* mutation could prevent: (1) the formation of DSBs; and/or (2) induce the rapid repair by homologous recombination using the sister chromatid as a substrate. The former idea is supported by a recent report in mice showing that axis localization of RAD51 foci and the DSB protein IHO1 is reduced in *Sycp2^–/–^* spermatocytes (Fujiwara et al., 2020). The latter is consistent with the conserved interaction of Red1/SYCP2 with HORMAD proteins (West et al., 2019), which promote inter-homolog bias (reviewed in Pradillo and Santos, 2011). Not surprisingly, based on the phenotypes in mice, homologous pairing is also impaired in *sycp2^–/–^* zebrafish spermatocytes (Takemoto et al., 2020). This would be because of the absence of homologous interactions mediated by Dmc1/Rad51 and/or defects in the SC formation as discussed above.

Sycp1 is also essential for zebrafish gametogenesis (Saito et al., 2011; Imai et al., 2021). In contrast to *sycp2^–/–^* zebrafish, signals of DSB marker proteins Dmc1/Rad51, RPA, and γH2AX are observed in *sycp1^–/–^* zebrafish spermatocytes (Imai et al., 2021). However, DSB repair is compromised in the *sycp1^–/–^* spermatocytes, and homologous pairing only transiently occurs at chromosome ends and is largely lost. Therefore, Sycp1 appears to function in later stages of homologous recombination to maintain alignment of homologs as observed with Sycp1 orthologs in other species (Zickler and Kleckner, 1999; de Vries et al., 2005; Higgins et al., 2005). Notably, in *sycp1^–/–^* spermatocytes, the majority of DSB marker signals appear at the proximity to telomeres on or adjacent to chromosome axes. This indicates that synapsis does not play a critical role in DSB localization to subtelomeres in zebrafish spermatocytes. Interestingly, Hormad1 persists on the axes in the absence of Sycp1, while bright Iho1 foci appear in leptotene and early zygotene-like spermatocytes and mostly disappear in the late zygote or pachytene-like stages, as in wild-type cells (Imai et al., 2021). In *Sycp1^–/–^* mice, IHO1 depletion from all axes has been observed in late zygotene to early pachytene stages (Dereli et al., 2021). How DSBs are directed to subtelomeric regions in zebrafish spermatocytes remains to be elucidated.

## Gonadal Sex Differentiation and Sex Reversal

In zebrafish, female development requires the continuous formation of oocytes, without which animals undergo female-to-male sex reversal (Kossack and Draper, 2019). Thus, mutations that disrupt oocyte development will cause animals to develop solely as males. This is feasible since male development does not depend on the presence of a Y chromosome. Mutations that knockout *spo11* or *mlh1* do not cause female-to-male sex reversal, possibly due to the absence of a synapsis checkpoint function absent in females as described in section “Is the Synapsis Checkpoint Absent During Oogenesis?”. As mentioned above, several mutations that affect the chromosome events of meiotic prophase also cause most or all of the homozygous mutant population to develop as males, including the axial component gene *sycp2*, the meiosis-specific cohesin subunits *smc1*β and *rad21l1*, and the DNA repair genes *brca2* and *fancl* (Rodríguez-Marí et al., 2010, 2011; Shive et al., 2010; Rodríguez-Marí and Postlethwait, 2011; Ramanagoudr-Bhojappa et al., 2018; Takemoto et al., 2020; Blokhina et al., 2021). One of our labs has reported that *sycp1^*isa*/*isa*^* populations develop solely as males (Imai et al., 2021), while the other has found females in a population of independently derived *sycp1^–/–^* mutants (Olaya and Burgess, unpublished). As sex is determined in zebrafish by both genetic and environmental factors, it is not surprising that they could also have epistatic effects on meiotic mutant phenotypes.

Tp53, the homolog of mouse and human TP53 plays dual roles in gonadal sex differentiation and as a checkpoint protein that can be activated by DNA damage. In a *brca2* mutant (*brca2^*Q*658X/Q658X^*) and *fancl^–/–^* zebrafish, mutation of *tp53* suppresses sex reversal and allows a portion of homozygous mutant animals to develop as females, yet these females produce malformed offspring due to aneuploidy (Rodríguez-Marí et al., 2010, 2011; Shive et al., 2010; Rodríguez-Marí and Postlethwait, 2011). These results suggest that the *brca2* and *fancl* mutations result in unrepaired DNA damage that would otherwise cause cells to be removed via a Tp53 mechanism.

In zebrafish, the mutant *rad21l1^–/–^* population also displays a dramatic shift in the sex ratio toward males due to late female-to-male sex reversal, and like *fancl* and *brca2* mutants, sex reversal can be partially suppressed in *tp53 rad21l1* double mutants (Blokhina et al., 2021). However, *rad21l1*^–/–^ zebrafish display exceptional sexually dimorphic phenotypes, with males producing healthy offspring and *tp53 rad21l1* females producing poor quality eggs and malformed embryos compared to *tp53* (Blokhina et al., 2021). This differs from the reproductive phenotype of *Rad21l^–/–^* mice where males are infertile and females display age-dependent sterility (Herrán et al., 2011). The fertility of *rad21l1*^–/–^ zebrafish may be due in part to the upregulation of the *rec8b* paralog (Blokhina et al., 2021). Interestingly, there are rare instances of *rad21l1^–/–^* female zebrafish with a normal reproductive phenotype, again another possible epistatic effect of the environment. The presence of lampbrush chromosomes at the follicle stage in 45 dpf animals demonstrates that these mutant oocytes can progress through meiosis prophase I to the diplotene stage but then undergo Tp53-mediated apoptosis, thus stimulating female-to-male sex reversal. The arrest is not likely due to the failure to repair Spo11-induced DSBs since the *spo11*^–/–^ *rad21l1*^–/–^ population develops as infertile males. This raises the possibility that Rad21l1 plays a role specific to oogenesis, either arising at or before the lampbrush stage.

## Is the Synapsis Checkpoint Absent During Oogenesis?

In zebrafish, the recombination mutants *mlh1*^–/–^ and *spo11*^–/–^ have similar phenotypes: in both strains, males are sterile and produce no sperm while mutant females are fertile, yet produce malformed progeny that fail to develop, likely due to severe aneuploidy (Feitsma et al., 2007; Leal et al., 2008; Blokhina et al., 2019). In *Mlh1^–/–^* and *Spo11^–/–^* mice, a significant number of oocytes form follicles after reaching the diplotene stage, yet accelerated loss of oocytes dramatically reduces the number of primordial follicles at the early postnatal period during follicle formation (Di Giacomo et al., 2005). The loss in oocytes has been attributed to a synapsis checkpoint that acts independently of a DSB repair checkpoint in mutants that fail to repair Spo11-DSBs. A similar synapsis checkpoint has been described in other model organisms (Roeder and Bailis, 2000; Bhalla and Dernburg, 2005; Subramanian and Hochwagen, 2014). The sexually dimorphic phenotypes associated with *mlh1*^–/–^ and *spo11*^–/–^ zebrafish suggest that a synapsis checkpoint may be absent during oogenesis but functional during spermatogenesis. These sexually dimorphic phenotypes may reflect infertility in humans. Azoospermia is a common phenotype associated with male infertility, while defects in the chromosome events of oogenesis tend to result in the formation of aneuploid embryos and is a major cause of pregnancy loss. Thus, infertility in human females may arise, in part, due to less robust checkpoint mechanisms (Nagaoka et al., 2012; Gray and Cohen, 2016). On the other hand, activation of meiotic checkpoints in male mammals can be traced to the meiotic silencing of unpaired chromosomes due to a disruption of the mechanism that accommodate the presence of unpaired sex chromosomes during normal meiosis (Turner et al., 2005). Notably, zebrafish do not have heteromorphic sex chromosomes, which enables the study of mutations affecting meiotic processes independent of the meiotic sex chromosome inactivation response (MSCI) (Burgoyne et al., 2009).

## Advantages and Disadvantages of the Zebrafish Model

In addition to the low cost in housing large numbers of animals (∼1/1000th the cost of mice), zebrafish share ∼70% of genes with humans and about 85% of human disease genes. Zebrafish have several unique features that make them a useful model organism to study the chromosome events of meiosis. First, progeny numbers in the hundreds and embryos develop outside of the body. The vast number of progeny generated in a single cross can account for large *n* values for reporting experimental results, on par with *Drosophila* and worm models. The transparency of the embryos enables researchers to pinpoint the stages of development that are affected by aneuploidy or chromosome abnormalities, whereas embryonic development is not as easily studied in mice. Second, oogenesis occurs over the course of adulthood in zebrafish, unlike in mice where the stages of meiotic prophase occur in the fetal ovary (discussed in section “Overview of Zebrafish Gametogenesis”). Thus, experiments involving the same female can be performed multiple times over the course of weeks. A single female can potentially breed 30 times. Third, sexually dimorphic phenotypes provide insights into meiotic errors. Maintenance of the ovary depends on the formation of oocytes, even into adulthood, and failure in oogenesis results in female-to-male sex reversal (discussed in section “Gonadal Sex Differentiation and Sex Reversal”). Therefore, defects in oogenesis can be assessed simply by assaying sex ratios in a tank of fish, not unlike the high incidence of male (*him*) phenotype in *C. elegans* that is used as a proxy for monitoring chromosome segregation errors (Hodgkin et al., 1979).

There are of course some downsides to working with zebrafish. The time to sexual maturity in zebrafish (∼60 days) is longer than in mice (∼35 days). While early gametogenesis can be studied in both species in a matter of weeks, the larval zebrafish gonads are so small it is difficult to obtain enough material to do chromosome spreads. This is especially challenging for studying mutants that undergo female-to-male sex reversal in the early larval stages. Conversely, the oocytes in adult fish contain a large amount of yolk, which is prohibitive for chromosome spreads. Development of meiocytes in these stages is not synchronous and is affected by environmental factors. Therefore, unlike mice, sampling of gonads enriched in a specific stage of prophase I is difficult. Since zebrafish have no sex chromosomes, processes that have evolved to accommodate unpaired sex chromosomes may be absent.

For the purposes of this review, the exceptional advantage to using zebrafish is the use of superresolution imaging to view the temporal events of meiotic prophase. While pairing occurs at several locations along a chromosome in many organisms, in a select few, pairing and the SC are established near the telomeres located in the bouquet, including human males. For this reason, zebrafish is a reasonable model to study sexually dimorphic features of human meiosis. Moreover, the isolation of pairing events to a specific region of the nucleus provides a window to examine how the crowding of chromosomes, in addition to chromosome movement, may support pairing. The possible role of bouquet in oocyte patterning is intriguing and should prove to be an interesting area of research. Any mechanism that supports pairing at the ends of chromosomes must also account for the removal of interlocks if chromosomes are to be properly synapsed at pachytene. Interlocks are commonly seen in chromosome spreads; it will be interesting to determine if there is a specific machinery to remove interlocks or entanglements. Since most chromosomes in spermatocytes have on average 1 crossover located at the end of the chromosomes, the loss of the SC at diplotene would naturally remove any interlocks. Thus, it is possible that interlock removal is not even necessary to progress through to anaphase (i.e., there is no interlock checkpoint) but this remains to be tested. The ability to easily see the coalignment of axes during the leptotene stage provides insight into the very early stages of pairing. It will be interesting to test if any epigenetic features of chromatin, or the chromosome axis, aid in homolog pairing. Axis proteins load at the telomeres and extend to the middle of the chromosome suggesting there may be an axis “seed” associated with telomere repeats. It is also unknown how/why telomeres of unrelated chromosomes associate with one another in the bouquet and if telomere-associated RNAs are involved. Another open question is whether the axis seeding and/or telomere-associated factors are involved in DSB induction at subtelomeric regions, potentially via recruitment of proteins essential for DSB formation.

## Future Perspectives

While zebrafish is a relative newcomer to the field of model organisms that are commonly used to study the dynamic chromosome events of meiosis, as a model, it has key features that are shared in common with human spermatogenesis that are less pronounced in other models; these include the positioning of DSBs, the initiation of pairing and synapsis of homologous chromosomes, and the skew of crossovers toward telomeres in the bouquet (Brown et al., 2005; Feitsma et al., 2007; Bisig et al., 2012; Pratto et al., 2014; Saito et al., 2014; Gruhn et al., 2016; Blokhina et al., 2019). Characterizing these features in zebrafish may provide insight into male infertility. Zebrafish is an important model for toxicology studies (Dai et al., 2014), providing the potential for doing research on chemicals that can affect gamete aneuploidy. In addition, *in vitro* culture systems are available for studying zebrafish meiosis. Recently, rapid chromosomal movements during early prophase I have been captured in culture juvenile zebrafish ovaries (Mytlis and Elkouby, 2021). The entire spermatogenesis process, from stem cell propagation to differentiation of functional sperm, has been recapitulated in culture (Sakai, 2002; Kawasaki et al., 2012, 2016). These resources are promising tools to investigate functions of meiotic chromosome dynamics along the absolute time axis by live and time-lapse imaging. Furthermore, forward-genetic screening approaches using ENU-mutagenesis identified mutations in *sycp2*, *sycp1*, and *mlh1* (Feitsma et al., 2007; Saito et al., 2011). The mutant phenotypes in gonadogenesis defects indicated that meiotic defects are the most common abnormalities. Continued genetic screening has the potential to identify additional meiotic actors. Genomic approaches to map the transcriptome of oocytes and spermatocytes, as has been done in mice, also has the potential to identify new genes with possible roles in meiosis (da Cruz et al., 2016; Geisinger et al., 2021). Teleost fish display remarkable diversity of reproductive strategies, though the underlying molecular mechanism remains largely unknown. Zebrafish studies will provide fundamental knowledge to approach these aspects in other fish species.

The basic framework for understanding the meiotic chromosome events of early to mid-prophase in zebrafish are coming into focus from studies reported in this review, including: (1) the analysis of key landmarks of meiotic prophase I in relationship to the bouquet in chromosome spreads at super-resolution imaging; (2) specifying the roles of key meiotic proteins Spo11, Sycp2, and Sycp1 and the cohesins Smc1β and Rad21l in processes related to the pairing, recombination and synapsis of homologous chromosomes; and (3) the development of methods for culturing and imaging live meiocytes. There are many unanswered questions and many opportunities for new researchers in the field.

## Author Contributions

YI and SB conceived the framework of the entire manuscript. YI, IO, NS, and SB wrote the manuscript. YI and IO prepared the figures. All the authors contributed to the article and approved the submitted version.

## Conflict of Interest

The authors declare that the research was conducted in the absence of any commercial or financial relationships that could be construed as a potential conflict of interest.

## Publisher’s Note

All claims expressed in this article are solely those of the authors and do not necessarily represent those of their affiliated organizations, or those of the publisher, the editors and the reviewers. Any product that may be evaluated in this article, or claim that may be made by its manufacturer, is not guaranteed or endorsed by the publisher.
